# 1581. Weight and Metabolic Changes with Long-Acting Lenacapavir in a Combination Regimen in Treatment-Naïve People with HIV-1 at Week 80

**DOI:** 10.1093/ofid/ofad500.1416

**Published:** 2023-11-27

**Authors:** Princy N Kumar, Deborah A Goldstein, Richard L Hengel, Aditya H Gaur, Anson K Wurapa, Ann M Khalsa, Cheryl L Newman, Gary Saunders, Shan-Yu Liu, Hadas Dvory-Sobol, Martin Rhee, Samir K Gupta

**Affiliations:** Georgetown University Medical Center, Potomac, Maryland; USAID, Washington DC, District of Columbia; Atlanta ID Group, Atlanta, Georgia; St. Jude Children's Research Hospital, Memphis, Tennessee; Infectious Disease Specialists of Atlanta, Decatur, Georgia; Valleywise Health, Phoenix, Arizona; Medical College of Georgia, Augusta, Georgia; Gilead Sciences Inc, Babraham, England, United Kingdom; Gilead Sciences Inc, Babraham, England, United Kingdom; Gilead Sciences, Foster City, California; Gilead Sciences, Foster City, California; Indiana University School of Medicine, Indianapolis, Indiana

## Abstract

**Background:**

Lenacapavir (LEN) is a highly potent, long-acting, first-in-class inhibitor of HIV-1 capsid protein approved for the treatment of HIV-1 infection in adults with multidrug resistance in combination with other antiretrovirals. CALIBRATE is an ongoing phase 2 study in people with HIV-1 (PWH) who are newly initiating treatment. At Week 80 (W80), subcutaneous (SC) and oral LEN, in combination with other antiretrovirals, maintained high rates of virologic suppression. In PWH initiating treatment, weight increases associated with a return to health effect have been observed. This analysis examined weight and metabolic changes to the W80 timepoint.

**Methods:**

Participants were randomized (2:2:2:1) to 1 of 4 treatment groups (TG). TG1 and TG2 both received SC LEN (927 mg) every 6 months + oral once daily (QD) emtricitabine/tenofovir alafenamide (F/TAF) for 28 weeks, after which virologically suppressed participants continued a 2-drug maintenance regimen: SC LEN (927 mg) with oral QD TAF (TG1) or oral QD bictegravir (BIC) (TG2). TG3 received oral QD LEN + F/TAF, and TG4 received oral QD BIC/F/TAF throughout. The metabolic profile of LEN was assessed from baseline to W28 and after initiating the 2-drug maintenance regimen to W80. Due to the small sample size, no statistical testing was performed.

**Results:**

182 participants (7% female, 52% Black) were randomized and dosed (n=52, 53, 52, 25 in TG1 to TG4, respectively). Baseline median age was 29 years; 15% had baseline viral load >100,000 c/mL. Baseline median weight and body mass index (BMI) were 78.2 kg and 25.8 kg/m^2^, respectively. Weight, BMI, and fasting lipid profiles for each treatment group through W80 are presented (Table).

**Figure:**
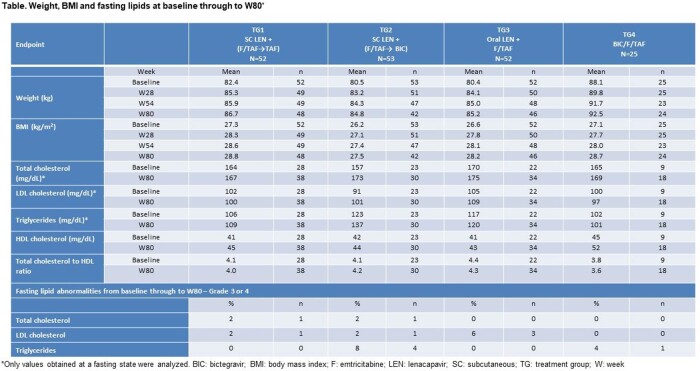

**Conclusion:**

In this phase 2 study of treatment-naïve PWH, treatment regimens that included SC or oral LEN in combination with other antiretroviral agents led to expected weight gain and increase in BMI, consistent with the return to health phenomenon, and were not associated with clinically relevant increases in lipids.

**Disclosures:**

**Princy N Kumar, MD**, Gilead Sciences, Inc: Grant/Research Support|Gilead Sciences, Inc: Stocks/Bonds|Johnson & Johnson: Advisor/Consultant|Johnson & Johnson: Stocks/Bonds|Merck: Advisor/Consultant|Merck: Grant/Research Support|Merck: Stocks/Bonds|Pfizer: Stocks/Bonds|Theratechnologies: Advisor/Consultant|Theratechnologies: Grant/Research Support|Viiv: Advisor/Consultant|Viiv: Grant/Research Support|Viiv: Stocks/Bonds **Aditya H Gaur, MD**, Gilead Sciences, Inc (Grant support/CTA with St. Jude): Grant/Research Support|Janssen (Grant support/CTA with St. Jude): Grant/Research Support|Viiv (Grant support/CTA with St. Jude and Serve on Pediatric Advisory Board): Board Member|Viiv (Grant support/CTA with St. Jude and Serve on Pediatric Advisory Board): Grant/Research Support **Anson K Wurapa, MD**, Gilead Sciences, Inc: Clinical Trial Investigator **Ann M. Khalsa, MD**, Gilead Sciences, Inc: Advisor/Consultant|Gilead Sciences, Inc: Grant/Research Support|Gilead Sciences, Inc: Honoraria|Glaxo Smith Kline: Advisor/Consultant|Viiv: Advisor/Consultant **Cheryl L Newman, MD**, Gilead Sciences, Inc: Grant/Research Support|GSK Viiv: Grant/Research Support|GSK Viiv: Honoraria|Janssen: Grant/Research Support|Merck: Grant/Research Support **Gary Saunders, BSc**, Gilead Sciences, Inc: Employee|Gilead Sciences, Inc: Stocks/Bonds **Shan-Yu Liu, PhD**, Gilead Sciences, Inc: Employee|Gilead Sciences, Inc: Stocks/Bonds **Hadas Dvory-Sobol, PhD**, Gilead Sciences, Inc: Employee|Gilead Sciences, Inc: Stocks/Bonds **Martin Rhee, MD**, Gilead Sciences, Inc: Employee|Gilead Sciences, Inc: Stocks/Bonds **Samir K. Gupta, MD**, Gilead Sciences: Advisor/Consultant|ViiV Healthcare: Advisor/Consultant|ViiV Healthcare: Grant/Research Support

